# Resolution of a bent-crystal spectrometer for X-ray free-electron laser pulses: diamond versus silicon

**DOI:** 10.1107/S2053273321003697

**Published:** 2021-05-27

**Authors:** Vladimir M. Kaganer, Ilia Petrov, Liubov Samoylova

**Affiliations:** a Paul-Drude-Institut für Festkörperelektronik, Leibniz-Institut im Forschungsverbund Berlin e. V., Hausvogteiplatz 5–7, 10117 Berlin, Germany; b European XFEL GmbH, Holzkoppel 4, 22869 Schenefeld, Germany

**Keywords:** X-ray free-electron lasers, X-ray spectroscopy, bent crystals, diamond crystal optics, femtosecond X-ray diffraction

## Abstract

The resolution function of a spectrometer based on a strongly bent single crystal (bending radius of 10 cm or less) is evaluated. It is shown that the resolution is controlled by two parameters: (i) the ratio of the lattice spacing of the chosen reflection to the crystal thickness and (ii) a single parameter comprising crystal thickness, its bending radius, distance to a detector, and anisotropic elastic constants of the chosen crystal. The results allow the optimization of the parameters of bent-crystal spectrometers for the hard X-ray free-electron laser sources.

## Abstract 2

The resolution function of a bent-crystal spectrometer for pulses of an X-ray free-electron laser is evaluated. Under appropriate conditions, the energy resolution reaches the ratio of the lattice spacing to the crystal thickness.

## 1. Introduction   

The self-amplified spontaneous emission (SASE) radiation pulses of an X-ray free-electron laser (XFEL) originate from random current fluctuations in the electron bunch (Saldin *et al.*, 2000 ▸). As a result, the intensity and the spectrum vary from one pulse to another. The spectral composition of every pulse used in an experiment needs to be measured individually.

**Figure 1:**
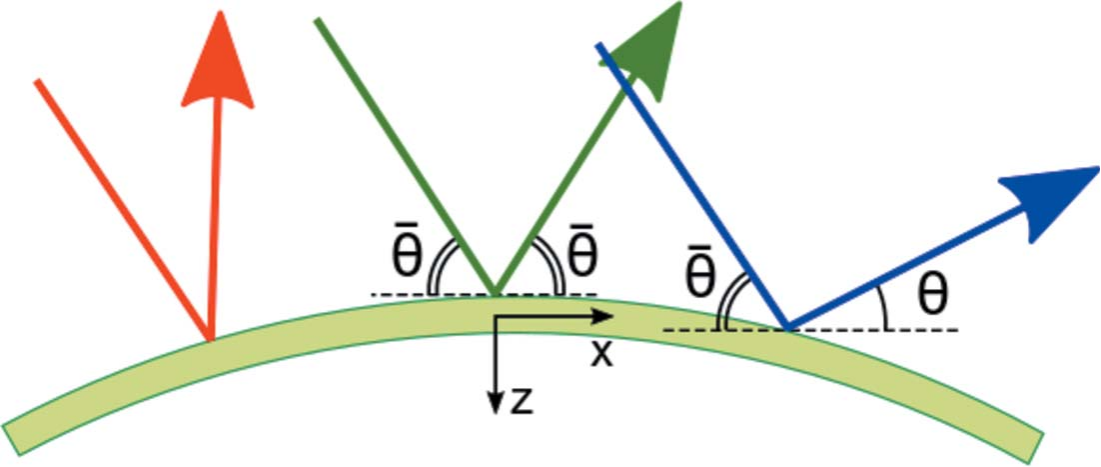
Schematic of a bent-crystal spectrometer. A collimated polychromatic beam is incident on a bent crystal. Each wavelength in the beam is diffracted at the position where the Bragg condition is satisfied for this wavelength. The scattering angle for a reference wavelength is 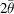 , while for another wavelength it is 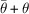 .

Diffraction at a bent crystal transforms the energy spectrum of a pulse into the angular spectrum of the diffracted waves, as shown in Fig. 1 ▸. Each wavelength in the initial polychromatic highly collimated X-ray pulse, incident on a bent crystal, finds a position on the crystal where the Bragg law is satisfied for this wavelength, and diffracts to the respective Bragg angle. Several bent-crystal spectrometers have been built and tested at the XFEL sources. Zhu *et al.* (2012 ▸), Rich *et al.* (2016 ▸) and Boesenberg *et al.* (2017 ▸) inserted bent crystals directly into the X-ray beam, while Makita *et al.* (2015 ▸) and Kujala *et al.* (2020 ▸) used the beams deflected by linear gratings for further diffraction at bent crystals.

To resolve the entire spectrum of a pulse, the bending radius needs to be chosen such that each wavelength in the spectrum finds a position at the bent crystal where its Bragg condition is satisfied. The Bragg conditions corresponding to a spectral width up to 100 eV have to be fulfilled within the spatial width of the X-ray pulse, which is below 1 mm at the XFEL sources. That requires bending radii of the order of 10 cm. Bending radii from 5 to 30 cm were used in the works cited above. A practical limit on the thickness of the bent crystal comes from the need to reach such small bending radii. The works cited above employed 10 µm-thick silicon or 20 µm-thick diamond plates in 110 and 111 orientations. Zhu *et al.* (2012 ▸) combined two bent-crystal spectrometers, with different bending radii and employing different reflections, one to cover the whole energy range and the other to reach a high resolution in a limited energy range.

Zhu *et al.* (2012 ▸) proposed that the resolution of the bent-crystal spectrometer is mainly limited by two different contributions originating from the dynamical diffraction parameters, the extinction depth Λ and the Darwin width 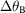 . The applicability of dynamical theory for planar crystals to the strongly bent ones was not argued, however. Boesenberg *et al.* (2017 ▸) and Kujala *et al.* (2020 ▸) considered the detector pixel size as a parameter limiting the resolution.

Recently, we have given a detailed theoretical description of the X-ray diffraction at strongly bent crystals (Kaganer *et al.*, 2020 ▸). We found that, because of the strong bending, the orientation of the crystal with respect to the incident X-ray wave deviates from the Bragg condition within the Darwin width 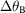 in an interval of distances that is small compared with the extinction length Λ. As a result, diffraction for bending radii smaller than the critical radius 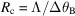 is kinematical. The kinematical diffraction approximation is satisfied in a broad range of crystal curvatures and X-ray energies. Spatial and angular distributions of the diffracted intensity have been calculated. In modelling diffraction of the XFEL pulses, it has been presumed that the scattering amplitudes for different frequencies add up coherently.

In the present work, we thoroughly analyse the diffracted intensity integrated over the pulse duration. We find that the coherent sum of the amplitudes describes an instant diffraction signal. In the time-integrated signal, the intensities, rather than the amplitudes, of different frequencies add up. Afanasev & Kohn (1977 ▸) arrived at a similar conclusion when analysing diffraction from a continuous incoherent X-ray source and averaging over random time instants of the emission of individual atoms.

We show that the spectral resolution of a bent-crystal spectrometer is controlled by two parameters. In the case of Fraunhofer diffraction, one parameter is simply the ratio of the lattice spacing of the actual reflection to the crystal thickness. The other parameter depends on the crystal thickness, its bending radius, and the anisotropic elastic constants of the crystal. These parameters are modified for finite crystal–detector distances (Fresnel diffraction). Still, two parameters controlling the resolution are derived.

## 2. Time-integrated diffraction intensity   

We assume full transverse coherence of the incident XFEL pulse and take into consideration only its time structure. The electric field of the pulse can be represented by its spectrum: 

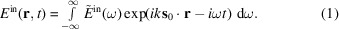

Here, ω is the frequency of a plane-wave component, 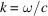 is its wavevector, *c* is the speed of light and 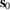 is the unit vector in the direction of the wave propagation.

The wave packet (1) is incident on a bent-crystal spectrometer. We consider its diffraction on a bent crystal in the kinematical (first Born) approximation. The applicability limits of this approximation were established by Kaganer *et al.* (2020 ▸) and are discussed in Section 5. We follow the description of the first Born approximation by Born & Wolf (2019 ▸, Section 13.1.2), but keep explicitly the time exponent 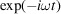 , which is usually implicit when considering diffraction of a monochromatic wave, and keep the integration over the frequencies ω. The amplitude of the scattered wave is 

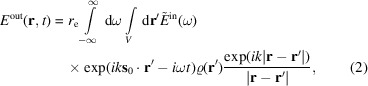

where 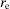 is the classical electron radius and 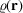 is the actual quantum density of the electrons in the crystal lattice [see also Landau & Lifshitz (1984 ▸), Section 124]. The spatial integration is performed over the volume *V* of the crystal.

In the Fraunhofer limit 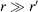 one has 

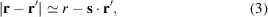

where 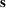 is the unit vector in the direction to the detector, 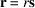 . The correction to equation (3) at smaller distances (Fresnel diffraction) is considered in Section 4. Using equation (3), the scattered wave (2) can be represented as 

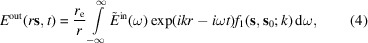

where the scattering amplitude in the first Born approximation is 

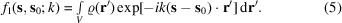

Here, the dependence of the scattering amplitude 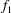 on the length of the wavevector *k* is explicitly noted.

The intensity of the scattered wave at the time instant *t* is 

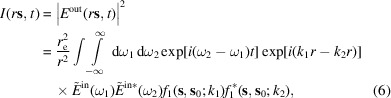

where the asterisk denotes the complex conjugate and 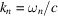 are the wavevectors ( 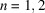 ).

We consider the measurement of the scattered intensity by a detector that integrates the intensity over the pulse duration (otherwise, if the detector were able to resolve the time structure of the pulse, the spectrometer would not be needed). Hence, the measured intensity is a result of integration over the pulse duration: 

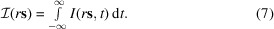

Integration of the time-dependent term in equation (6) gives rise to a delta function 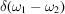 , so that the intensity integrated over the time is 

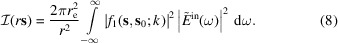

This equation replaces equation (17) of Kaganer *et al.* (2020 ▸), where a coherent superposition of the waves with different wavevectors has been presumed.

The squared scattering amplitude 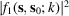 in equation (8) is a function of the frequency ω and the scattering angle (the angle between vectors 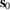 and 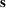 ). One can consider the scattering angle as twice the Bragg angle for another frequency 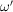 , which is defined by this condition. Then, the scattering intensity is represented as a function of 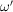 . This representation is used in all papers on the bent-crystal spectrometers cited in Section 1 since it allows one to conveniently present the angular spectrum of the scattered intensity in the same scale as the energy spectrum of the incident pulse. Explicit expressions relating the scattering angle to the artificial frequency 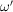 are derived in the next section. Here we denote 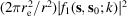 as 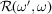 and rewrite equation (8) as an integral: 

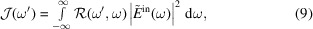

where 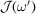 is the intensity (8) after the change of variables from θ to 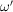 . One can see that if the resolution is ideal, *i.e.* 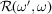 is 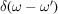 , the spectrum of the diffracted waves in the 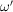 scale coincides with the spectrum of the incident wave. The aim of the next sections is to calculate the resolution function 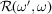 for a bent-crystal spectrometer.

## 3. Resolution of a bent-crystal spectrometer: Fraunhofer diffraction   

The scattering amplitude (5) for an ideal crystal is governed by the Fourier component 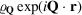 of the electron density 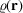 for the actual reciprocal-lattice vector 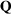 . The displacement field 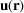 due to the bending causes the change of the electron density according to the change of the positions of the atoms to 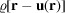 and the respective change of its Fourier component to 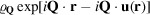 .

The kinematical diffraction amplitude (5) can be written as an integral over the scattering plane of the crystal:

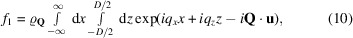

where *D* is the thickness of the crystal plate and 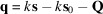 . Here, the scattering plane is the *xz* plane with the *x* axis tangent to the surface of the bent crystal at 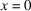 and the *z* axis along the inner surface normal. The origin is taken in the middle plane of the crystal plate (see Fig. 1 ▸). Let us calculate the components of the scattering vector 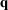 in this frame.

An X-ray pulse consists of plane waves of different frequencies incident onto the crystal at the same angle 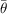 with respect to the *x* axis. The angle 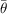 is the Bragg angle for some reference frequency 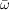 with the wavevector 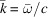 . The Bragg law for this frequency reads 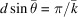 , where 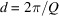 is the lattice spacing of the chosen reflection. The *x* and *z* components of the reciprocal-lattice vector in the chosen coordinate system are 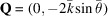 .

The wavevector of the incident wave with any other frequency ω in the pulse possesses the same incidence angle 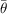 but another wavevector *k*, so that 

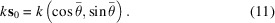

The diffracted intensity is measured as a function of the angle θ between the *x* axis and the vector 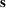 (see Fig. 1 ▸). Hence, the wavevector of the diffracted wave is 

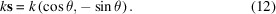

As mentioned in the previous section, it is convenient to consider the scattering angle 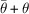 as twice the Bragg angle of a wave with the frequency 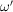 defined by this condition. The Bragg law reads 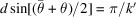 , where 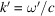 is the respective wavevector. A straightforward calculation of the components of the vector 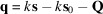 gives [see also Appendix B by Kaganer *et al.* (2020 ▸)] 

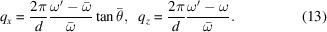

For symmetric Bragg reflections considered in the present work, 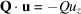 , so that only the 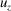 component of the displacement field is of interest. For a crystal cylindrically bent to a radius *R*, it is (Kaganer *et al.*, 2020 ▸) 

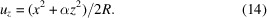

To achieve the cylindrical bending of a rectangular plate, the bending momenta have to be applied to the perpendicular edges of the plate. The same bending state can be approached by applying a momentum to the apex of a triangle-shaped plate (Terentyev *et al.*, 2016 ▸).

The parameter α in equation (14) depends on the anisotropic elastic constants of the crystal, particularly, for a 110-oriented diamond plate, 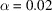 , while for a silicon plate of the same orientation 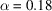 . For 111-oriented plates, the respective values are 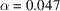 for diamond and 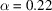 for silicon. Exceptionally small values for diamond are a result of compensation of the Poisson and anisotropy effects, which gives rise to a little depth dependence of the lattice spacing.

The term 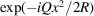 in the integral (10) due to the displacement field of a bent crystal gives rise to an *x* range relevant to diffraction of the order of 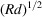 , which is comparable with *D* and much smaller than the lateral dimensions of the crystal. Hence, the integration over *x* in equation (10) can be performed in infinite limits. This integration results in a phase factor that drops out when calculating 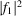 . In the remaining integral over *z*, we proceed to a dimensionless variable 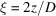 . Then, the integral (10) gives 

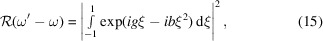

where it is denoted 

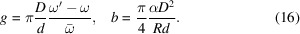

In the case of Fraunhofer diffraction, only the 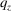 component of the scattering vector in equation (13) is relevant. The resolution function in equation (9) depends on the difference 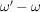 , so that we write it as 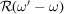 , and the integral (9) is a convolution integral.

Before calculating the integral (15), let us discuss its properties qualitatively. As long as the parameter *b* is small enough, the second term in the exponent in equation (15) can be neglected. Numerical examples presented below show that, practically, *b* does not need to be very small: this term can be neglected for 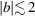 . When this condition is satisfied, the resolution function is simply 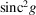 , where 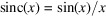 . We write hereafter 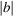 , where this is relevant, since the radius *R* can be positive for a convex crystal bending, as shown in Fig. 1 ▸, or negative for a concave bending.

The condition 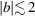 imposes a lower limit to the radius *R*. At the same time, the upper limit to the radius is the requirement of the applicability of the kinematical approximation, so that the radius should be small compared with the critical radius 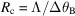 . The theoretical examples presented in this section and the experimental examples in Section 5 show that the interval between these limits is broad enough and covers a practically feasible range of parameters.

The resolution can be quantified using the Rayleigh criterion, formulated for spectral lines with the shapes described by the function 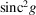 (see Born & Wolf, 2019 ▸, Section 7.6.3). Rayleigh proposed that two components of the same intensity are just resolved, when the principal intensity maximum of one coincides with the first intensity minimum of the other. This criterion corresponds to 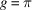 and gives the resolution 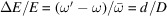 . Hence, provided 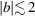 , the relative resolution 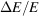 does not depend on the X-ray energy and on the bending radius and is equal to the ratio of the lattice spacing of the chosen reflection to the thickness of the bent crystal.

The integral (15) can be calculated and expressed through cosine and sine Fresnel integrals 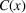 and 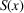 as 

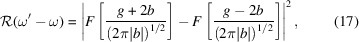

where 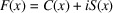 .

**Figure 2:**
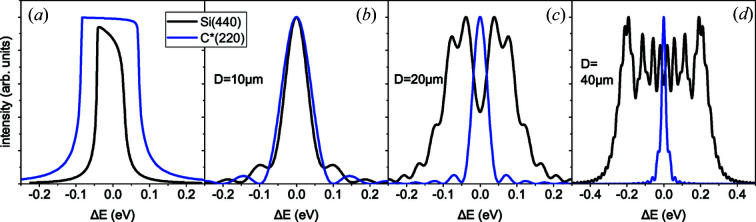
Fraunhofer diffraction curves for Si(440) and C*(220) reflections at the X-ray energy 8 keV: (*a*) Darwin curves, (*b*)–(*d*) diffraction from crystals bent to a radius of 0.1 m, at crystal thicknesses (*b*) 10 µm, (*c*) 20 µm and (*d*) 40 µm calculated by equation (17).

Fig. 2 ▸ compares the Fraunhofer diffraction curves for Si(440) and C*(220) reflections at the X-ray energy of 8 keV. Fig. 2 ▸(*a*) compares Darwin curves, *i.e.* the diffraction curves for non-bent infinitely thick crystals. The Darwin widths are 0.07 and 0.15 eV for silicon and diamond reflections, respectively. Figs. 2 ▸(*b*)–2 ▸(*d*) compare the diffraction curves, calculated by equation (17), from the crystal plates of different thicknesses bent to a radius of *R* = 0.1 m.

The curves in Fig. 2 ▸(*b*) are calculated for the crystal thickness of *D* = 10 µm. The values of the parameter *b* given by equation (16) are 1.47 and 0.125 for silicon and diamond reflections, respectively. Both values are smaller than 2, the shape of the resolution functions is given by 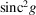 and gives rise to the resolutions 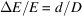 . The diamond reflection provides a slightly worse resolution, since the lattice spacing is larger. Fig. 2 ▸(*c*) shows diffraction curves for a two times larger thickness, *D* = 20 µm. The values of the parameter *b* increase by a factor of 4. As a result, this parameter becomes larger than 2 for silicon ( 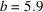 ), which results in a substantial broadening of the diffraction line. This parameter remains smaller than 2 for diamond ( 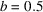 ), and the resolution improves by a factor of 2 compared with Fig. 2 ▸(*b*), since the crystal thickness is two times larger. In Fig. 2 ▸(*d*), the thickness is again increased by a factor of 2, to *D* = 40 µm. The factor *b* becomes four times larger compared with the case of Fig. 2 ▸(*c*). For silicon, it is large, 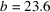 , and the diffraction curve broadens further. For diamond, it just reaches the value 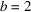 , and the resolution improves by a factor of 2 and reaches the value 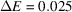 eV. Further increase of the thickness causes a reduction in the resolution for diamond as well. We note that the resolution obtained for a 40 µm-thick strongly bent diamond plate is six times better than that of a planar crystal given by its Darwin width.

Thus, the recipe to get the best resolution in Fraunhofer diffraction from a strongly bent crystal is to maximize the ratio 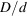 while keeping the parameter *b* in equation (16) smaller than 2. The exceptionally small value of α for diamond allows larger thicknesses, while keeping 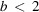 , and results in a notably better resolution. This recipe is modified at finite distances to a detector, which is discussed in the next section.

**Figure 3:**
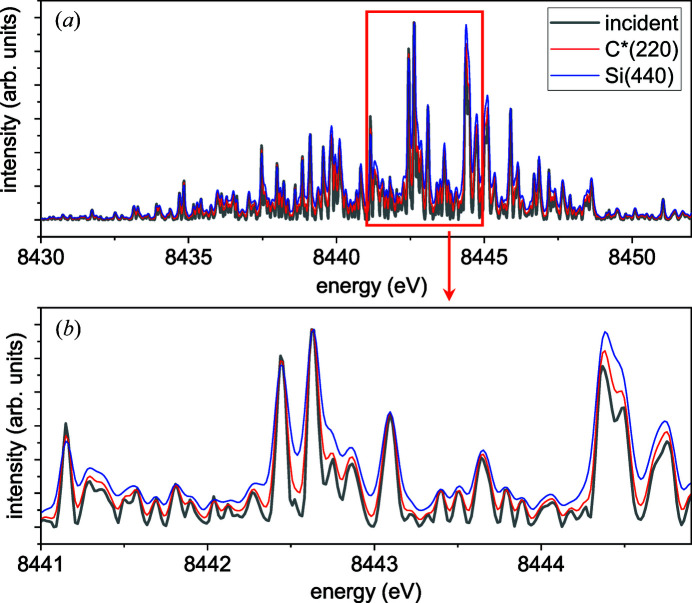
Spectrum of a SASE pulse incident on the bent-crystal spectrometers (grey lines) and spectra of the diffracted waves for the C*(220) reflection from a 20 µm-thick crystal (red lines) and for the Si(440) reflection from a 10 µm-thick crystal (blue lines), both bent to a radius of 0.1 m. Fraunhofer diffraction. (*a*) Whole spectrum, (*b*) magnified central part.

Fig. 3 ▸ compares a spectrum of the XFEL pulse incident on the bent crystal with the calculated spectra obtained with the bent-crystal spectrometers. The pulse generated during the SASE process at the European XFEL is simulated with the code *FAST* (Saldin *et al.*, 1999 ▸), which provides a 2D distribution of electric field in real space at the exit of the undulator for each time moment for various parameters of the electron bunch and the undulator. Simulation results are stored in an in-house database (Manetti *et al.*, 2019 ▸). The pulse is simulated for the electron energy 17.5 GeV and the active undulator length corresponding to the low-gain regime of SASE (Schneidmiller & Yurkov, 2014 ▸). Conversion from the time domain to the frequency domain is performed using the *WavePropaGator* package (Samoylova *et al.*, 2016 ▸), which provides a 2D distribution of electric field for each frequency of the pulse. We use the spectrum at the centre of the pulse in the frequency domain, assuming this distribution to be the same across the beam.

The spectra obtained with the bent-crystal spectrometers are calculated by equation (9) with the resolution function (17). Two reflections from crystals bent to a radius *R* = 0.1 m are compared: Si(440) from a 10 µm-thick plate and C*(220) from a 20 µm plate. The latter gives two times better resolution compared with the former, *cf*. Figs. 2 ▸(*b*), 2 ▸(*c*). A characteristic separation between the peaks in the spectrum is 0.12 eV. These peaks are well resolved by the C*(220) spectrometer with its resolution of 0.048 eV and still resolved with the Si(440) spectrometer with the resolution of 0.08 eV.

## 4. Resolution of a bent-crystal spectrometer: Fresnel diffraction   

This section is devoted to evaluating the effect of a finite distance between the bent-crystal spectrometer and a detector. The finite distance to a detector is accounted for by the subsequent term in the expansion (3): 

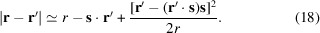

Substituting 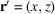 and 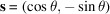 in the coordinates of Fig. 1 ▸, we find that equation (10) acquires an additional phase term 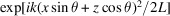 in the integral. Hereafter, the spectrometer–detector distance is denoted by *L* instead of *r* for convenience. The wavevector *k* and the scattering angle θ can be replaced in this term with sufficient accuracy with the reference values 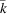 and 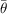 .

Calculation of the integral (10) with this additional phase factor has been performed in our previous work (Kaganer *et al.*, 2020 ▸). After evaluation of the integral over *x* in the infinite limits, we arrive at a modified equation (15), 

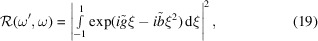

where 

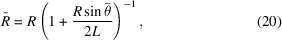




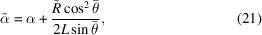




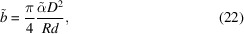




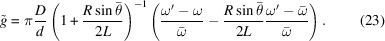

Calculation of the integral (19) is the same as in equation (17) above: 

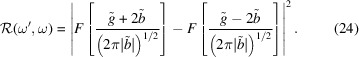

The resolution function in equation (24) depends on ω and 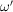 , rather than the difference 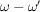 in equation (17). As a result, the diffracted spectrum is stretched or squeezed, depending on the sign of the bending. This is discussed below.

Similarly to the Fraunhofer diffraction case described in the previous section, a maximum resolution is reached as long as 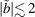 . If this condition is satisfied, the resolution according to the Rayleigh criterion, which follows from equation (23), is 

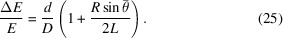

If 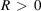 , in the case of the convex bending shown in Fig. 1 ▸, the resolution becomes worse compared with the Fraunhofer diffraction. Conversely, for a concave bending 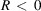 , the resolution is better than in the case of Fraunhofer diffraction. The correction can be notable when the bending radius *R* and the distance to a detector *L* are comparable. We expect, however, that 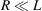 is a more common case. We take into account this correction in the numerical calculations below but do not discuss it further.

The parameter 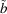 in equation (22) can be rewritten as 

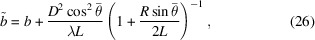

where λ is the wavelength, and the Bragg law 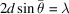 is used. Kaganer *et al.* (2020 ▸) considered this parameter in the case 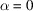 (and hence 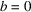 ) and stated that the Fraunhofer limit is reached when the diameter of the first Fresnel zone 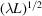 exceeds the crystal thickness seen from the direction of the diffracted beam, 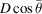 . In the general case 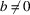 , both contributions to 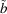 need to be taken into account, to provide the desired condition 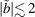 .

**Figure 4:**
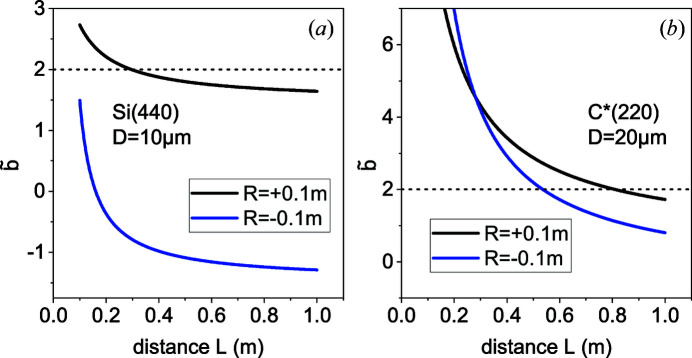
The dependence of the parameter 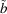 given by equation (22) on the distance *L* to a detector for the X-ray energy of 8 keV, bending radii 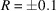 m, and (*a*) Si(440) reflection at 10 µm crystal thickness and (*b*) C*(220) reflection at 20 µm crystal thickness.

Since the sign of *b* coincides with the sign of *R*, the condition 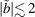 is satisfied at different distances *L* for the convex and the concave bending. The effect of finite distances is especially strong for diamond, since the parameter α, and hence *b*, are especially small. As an illustration, Fig. 4 ▸ presents the dependencies of the parameter 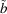 on the distance to a detector *L* at the X-ray energy of 8 keV for the bending radii 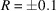 m. We choose a 10 µm-thick silicon and a 20 µm-thick diamond since such crystal thicknesses have been used in the experimental works discussed in the next section.

Fig. 4 ▸(*a*) shows the parameter 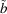 for a 10 µm-thick silicon plate in the reflection (440). At a distance *L* = 1 m, the values of 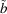 are close to the Fraunhofer limit 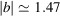 . For a convex bending, the value of 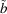 remains smaller than 2, and hence a maximum resolution is provided, down to a distance of 0.3 m. For a concave bending, 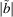 remains smaller than 2 for distances exceeding 0.1 m, as a result of the compensation of the curvature of the lattice planes and the curvature of the wavefront.

Fig. 4 ▸(*b*) shows a corresponding calculation for a 20 µm-thick diamond plate in the reflection (220). A notable deviation from the value 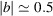 in the Fraunhofer limit takes place already at a distance of 10 m. A critical value 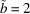 is reached at a distance of 0.8 m for a convex bending and at a distance of 0.55 m for a concave bending. As expected, finite distances to a detector give rise to a stronger effect for diamond since the finite distance correction in equation (26) is added to a smaller *b*.

**Figure 5:**
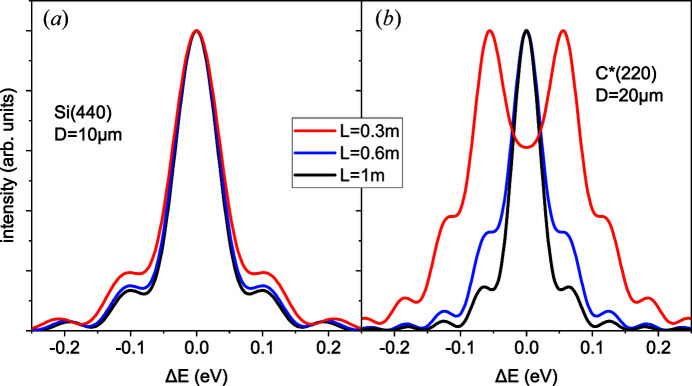
Resolution curves for Fresnel diffraction at the X-ray energy of 8 keV for (*a*) Si(440) reflection, crystal thickness 10 µm, and (*b*) C*(220) reflection, crystal thickness 20 µm. Bending radius *R* = 0.1 m, distances to a detector 0.3, 0.6 and 1 m.

Fig. 5 ▸ compares resolution functions for the same diffraction conditions as in Fig. 4 ▸ at different distances to a detector and the convex crystal bending, *R* = 0.1 m. When the distance *L* is reduced, the resolution curves calculated for Si(440) in Fig. 5 ▸(*a*) change only a little, in agreement with Fig. 4 ▸(*a*), where the value of 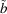 remains smaller than 2 for the distances *L* exceeding 0.3 m. In contrast, the resolution curves for C*(220) in Fig. 5 ▸(*b*) are notably modified and broadened since 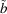 in Fig. 4 ▸(*b*) increases with the reducing distance *L* and exceeds the value of 2 for *L* < 0.8 m. We note that Figs. 4 ▸ and 5 ▸ compare silicon and diamond crystals of different thicknesses. For a silicon crystal of the same thickness of 20 µm as diamond, the value of 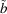 would increase by a factor of 4, and the desirable resolution by equation (25) could not be reached.

**Figure 6:**
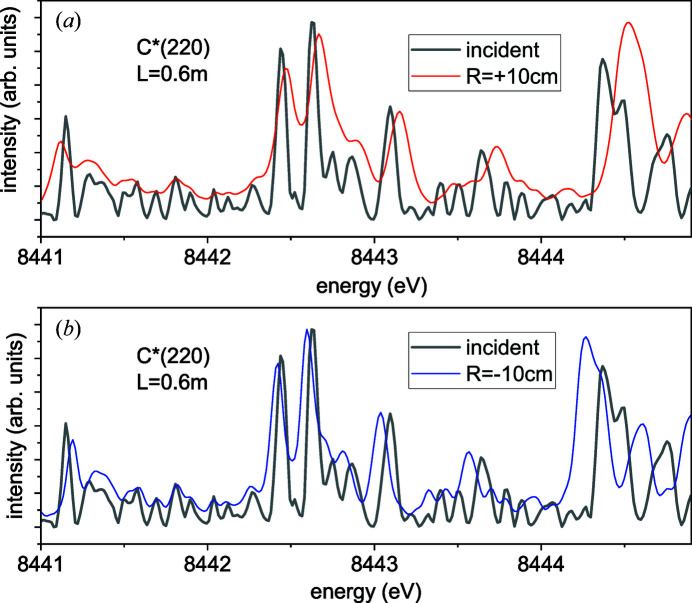
A selected region of a SASE pulse spectrum, the same as in Fig. 3 ▸(*b*), and the spectra of the diffracted waves for the C*(220) reflection from *D* = 20 µm crystal and the distance from the bent-crystal spectrometer to a detector *L* = 0.6 m. (*a*) Convex bending, *R* = +0.1 m, (*b*) concave bending, *R* = −0.1 m.

Fig. 6 ▸ shows the same selected region of a SASE pulse spectrum as in Fig. 3 ▸(*b*), and the spectra of the diffracted waves calculated by equation (9) with the resolution function (19) for the C*(220) reflection from a 20 µm-thick crystal bent to radii 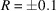 m at the distance to a detector of *L* = 0.6 m. At this distance, as follows from Fig. 4 ▸(*b*), the parameter 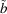 is slightly larger than 2 for the convex bending and somewhat smaller than 2 for the concave bending. As a result, the diffracted wave spectrum for the concave bending in Fig. 6 ▸(*b*) reveals somewhat better resolution compared with the convex bending case in Fig. 6 ▸(*a*).

The diffracted spectra in Fig. 6 ▸ are scaled with respect to the incidence spectrum. The spectrum is stretched for 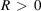 and squeezed for 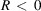 . This effect reduces with the increasing distance to a detector and vanishes in the Fraunhofer limit. As discussed in Section 3, the curves of diffracted intensity are not a real spectrum. Rather, they are a representation of the angular distributions of the intensity in terms of energy distributions. For Fraunhofer diffraction, such recalculation of the angular spectrum differs from the incident spectrum because of the finite resolution. At finite distances between the bent-crystal spectrometer and a detector, the diffracted spectrum is also scaled with respect to the incident spectrum.

## 5. Discussion   

Table 1 ▸ compares the resolutions of the bent-crystal spectrometers, reported in the experimental studies, with the calculated resolutions. The accuracy in determination of both quantities is limited by a number of factors. In the experiments, the spectrum of the incident pulse is not known. The resolution inferred from the oscillations in the diffracted spectrum can provide only an upper bound for the resolution since a resolution better than the widths of the oscillations will not show up. An additional smearing of the diffracted intensity is caused by a detector resolution, given by its pixel size and a finite distance to it.

In the calculations, even the diffraction of a monochromatic incidence wave gives rise to complicated intensity profiles like the ones shown in Figs. 2 ▸ and 5 ▸, which need to be characterized by a single number representing the resolution. The full width at half-maximum (FWHM) of the calculated diffraction curves seems a reasonable characteristic of the curves, although it ignores their internal structure. However, we would like to keep the resolution given by the Rayleigh criterion for the cases of the best available resolution, when the resolution function is given by 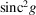 , as in Figs. 2 ▸(*b*) and 5 ▸(*a*). In these cases, the Rayleigh resolution is 1.13 times larger than the FWHM of the respective peak. Therefore, we define the resolution 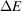 as 1.13 × FWHM. The values thus obtained from the intensity curves calculated by equation (24) are presented in Table 1 ▸ for the respective experimental conditions.

As a first step of the analysis, we calculate the critical radii 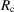 for the applicability of the approximation of kinematical diffraction employed in the present work. The critical radius is given by the ratio 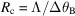 of the Bragg case extinction length Λ to the Darwin width 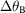 (Kaganer *et al.*, 2020 ▸). If the bending radius is small compared with the critical radius, the length of the path of the incident wave in the crystal, where it remains in conditions of dynamical diffraction, *i.e.* within the angular range 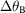 , is small compared with the length Λ needed to produce a strong diffracted wave. Then, the diffracted wave remains weak compared with the transmitted wave and can be calculated kinematically. Both quantities, Λ and 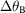 , are calculated using Sergey Stepanov’s *X-Ray Server* (Stepanov, 2004 ▸, 2021 ▸) for respective energies and reflections, and the resulting critical radii 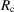 are presented in Table 1 ▸.

The bending radii *R* are smaller than the respective critical radii 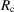 in all cases listed in Table 1 ▸, except for the Si(111) reflection reported by Zhu *et al.* (2012 ▸). This reflection was chosen to cover the whole spectrum of the pulse at the cost of lower resolution. We calculate the energy resolution in the kinematical approximation for this case as well, for the sake of completeness.

Next, we calculate, for every experiment listed in Table 1 ▸, the parameter 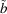 by equation (22). As long as 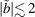 , the optimum resolution given by equation (25) is reached. The Si(220) reflection by Rich *et al.* (2016 ▸) corresponds to a large value 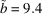 and gives rise to a low energy resolution. In all other cases, 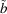 does not exceed 3.6, and the resolution is worse than the optimum one, up to a factor 3.4.

The calculated energy resolutions 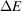 presented in Table 1 ▸ are obtained by simulating the respective resolution curves by equation (24) and numerically obtaining the FWHMs of the curves. The resolution is defined to be 1.13 × FWHM, as suggested above. In all cases, the calculated resolutions in Table 1 ▸ are smaller than the corresponding experimental resolutions. This is expected since our calculations do not take into account a finite detector resolution, partial incoherence of the SASE pulse, the X-ray amplitude variation across the pulse, and further possible inhomogeneities in the experiments.

The energy resolution of a bent-crystal spectrometer is given mostly by the ratio 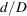 . The finite-distance correction factor 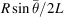 in equation (25) is about 0.1 or less for the spectrometers in Table 1 ▸. To improve the resolution, one would need to increase the crystal thickness *D*. However, there is almost no room for that since 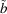 is proportional to 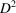 and should not exceed 2, while in Table 1 ▸ it is at that limit or above in most cases. A possible way to increase the silicon crystal thickness is to use concave bending, 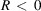 . Then, the two terms in equation (21) have different signs and partially compensate each other. For diamond with its small α, an increase of the distance to a detector *L* allows one to increase *D* and hence increase the resolution. For a large crystal–detector distance, as exemplified in Fig. 2 ▸(*d*), a resolution 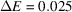 eV, or 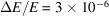 , can be obtained at a thickness of the diamond crystal of 40 µm.

The general formulas are illustrated in Sections 3 and 4 by calculations at an X-ray energy of 8 keV since this value is close to the energies in the experiments listed in Table 1 ▸. In the case of Fraunhofer diffraction, the parameter *b* in equation (16) and the relative energy resolution 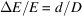 are energy independent. However, at higher X-ray energies, one can proceed to higher reflection orders. If other parameters are kept unchanged, the parameter *b* increases ( 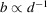 ) and may exceed the value of 2, which will result in a loss in resolution. As soon as *b* is smaller than 2, the absolute resolution 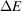 remains the same in higher reflection orders.

In the case of Fresnel diffraction, the parameter 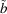 in equation (22) and the energy resolution (25) depend on the X-ray energy through the variation of the Bragg angle 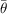 . This effect seems minor, at least for 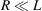 . Let us take a two times greater X-ray energy of 16 keV for a comparison. Then, the second orders of the reflections considered in Section 4, namely Si(880) and C*(440), can be used and give just the same Bragg angles as the ones in reflections Si(440) and C*(220) at 8 keV. If other parameters are not changed, the value of 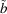 increases by a factor of 2. It follows from Fig. 4 ▸ that at the convex bending the values of 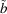 will be larger than 3, which results in a decrease in resolution. For concave bending, the diamond crystal can be still used without a loss in resolution for the distances *L* to a detector of 1 m or more, while the silicon crystal can be used at smaller distances.

## 6. Conclusions   

We have shown that the angular distribution of the intensity diffracted by a bent crystal and integrated over the pulse duration is given by a convolution of the spectrum of the incident X-ray pulse with the resolution function of the bent-crystal spectrometer. This result does not depend on the degree of temporal coherence.

We have evaluated the resolution of the bent-crystal spectrometer. It is controlled by two parameters. One parameter is the ratio 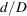 of lattice spacing of the chosen reflection to the thickness of the bent crystal. This ratio is the best achievable resolution, 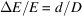 . It is reached if 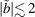 , where the parameter 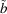 given by equation (22) combines in a single parameter the crystal thickness *D*, the curvature radius *R*, the distance to a detector *L*, the lattice spacing of the actual reflection *d*, and the parameter α representing the anisotropic elastic properties of the crystal. As an example, for the C*(220) reflection and a crystal thickness of 40 µm, the resolution of 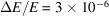 can be reached. These results allow one to optimize the parameters of the bent-crystal spectrometers for the XFEL radiation pulses.

## Figures and Tables

**Table 1:** Resolutions of the bent-crystal spectrometers reported in the experiments and the corresponding calculated resolutions The critical radius R_{\rm c} for applicability of the kinematical calculation is given by the ratio of the extinction length to the Darwin width \Lambda/\Delta\theta_{{\rm B}}. Bending radii *R*, crystal thicknesses *D* and the distances *L* to a detector are taken from the corresponding publications. The parameter \tilde{b} is calculated by equation (22). The calculated resolution \Delta E is obtained from the FWHMs of the intensity curves calculated by equation (24). The experimental resolutions are the ones reported in the corresponding publications.

								Resolution	
Reference	Reflection	Energy (eV)	Critical radius R_{\rm c} (m)	Bending radius *R* (m)	Thickness *D* (µm)	Distance *L* (m)	\tilde{b}	Calculated \Delta E (eV)	Experiment \Delta E (eV)
Zhu *et al.* (2012 ▸)	Si(111)	8330	0.05	0.30	10	0.33	1.55	0.30	0.5
	Si(333)	8330	0.96	0.15	10	0.44	1.63	0.10	0.13
Makita *et al.* (2015 ▸)	Si(333)	8330	0.96	0.078	10	0.33	2.85	0.14	0.30
	Si(220)	8330	0.09	0.05	10	0.2	3.61	0.57	–
Rich *et al.* (2016 ▸)	Si(440)	8330	0.60	0.10	10	0.5	1.86	0.09	0.15
	Si(220)	8330	0.09	0.05	20	0.5	9.41	0.83	–
Boesenberg *et al.* (2017 ▸)	C*(220)	7610	0.25	0.10	20	0.35	3.44	0.17	0.24
	C*(440)	10500	1.71	0.10	20	0.35	1.82	0.04	0.32
	Si(220)	7610	0.08	0.05	10	0.27	2.88	0.24	0.61
	Si(440)	7610	0.46	0.05	10	0.27	3.41	0.26	0.32
Kujala *et al.* (2020 ▸)	Si(220)	9310	0.10	0.10	10	1	1.25	0.19	0.55
	Si(440)	9310	0.77	0.10	10	1	1.77	0.10	0.15
	Si(333)	9310	1.19	0.075	10	1	2.55	0.12	0.15
	C*(220)	9310	0.35	0.125	20	1	2.05	0.07	0.25
